# Comparison of the utility of SARC-F, SARC-CalF, and calf circumference as screening tools for sarcopenia in patients with osteoporosis

**DOI:** 10.1371/journal.pone.0310401

**Published:** 2024-10-22

**Authors:** Yuki Ishihara, Toru Kusakabe, Akihiro Yasoda, Takuya Kitamura, Kazutaka Nanba, Mika Tsuiki, Noriko Satoh-Asahara, Tetsuya Tagami

**Affiliations:** 1 Department of Endocrinology and Metabolism, National Hospital Organization Kyoto Medical Center, Kyoto, Japan; 2 Department of Endocrinology, Metabolism, and Hypertension Research, Clinical Research Institute, National Hospital Organization Kyoto Medical Center, Kyoto, Japan; 3 Clinical Research Institute, National Hospital Organization Kyoto Medical Center, Kyoto, Japan; Graduate School of Medicine, Kyoto University, JAPAN

## Abstract

**Aim:**

Patients with osteoporosis who also have sarcopenia are at a high risk for falls and fractures. Early detection of sarcopenia is crucial for these patients. This study aimed to compare the effectiveness of SARC-F, SARC-CalF, and calf circumference (CC) as screening tools for sarcopenia in patients with osteoporosis.

**Methods:**

This cross-sectional study was retrospectively conducted on patients who attended the outpatient clinic for Osteoporosis and Sarcopenia at Kyoto Medical Center. Sarcopenia was determined based on low skeletal muscle mass and weak handgrip strength. Sensitivity and specificity analyses were conducted on SARC-F, SARC-CalF, and CC. The diagnostic utility of these three tools was compared using the receiver-operating characteristic (ROC) curves and the area under the ROC curves (AUC).

**Results:**

A total of 225 patients (men/women: 33/192) with a median age of 69.0 years (interquartile range: 61.0‒75.0) were enrolled. The prevalence of sarcopenia was found to be 11.6%. CC had the highest sensitivity (80.8%), while SARC-F had the highest specificity (93.0%) for detecting sarcopenia. ROC analysis revealed that all three tools had significant potential for sarcopenia diagnosis, with SARC-CalF having the highest AUC compared to SARC-F and CC (0.753 *vs*. 0.619 and 0.700). A multivariate logistic regression, incorporating other confounders as explanatory variables, revealed that SARC-CalF was independently related to sarcopenia (odds ratio: 14.80, 95% confidence interval: 3.83–57.30, *p* < 0.001).

**Conclusion:**

In patients with osteoporosis, SARC-CalF is more effective in the early detection of sarcopenia than SARC-F and CC.

## Introduction

In an aging society, the extension of healthy life expectancy is an urgent issue, necessitating early detection and treatment of diseases that cause elderly people to require nursing care [1]. Both osteoporosis and sarcopenia are age-related diseases that significantly increase the risk of falls and fractures, resulting in a decline in activities of daily living and quality of life [2].

Osteoporosis is characterized by the loss of bone mineral density (BMD) and the micro-architectural deterioration of bone structures, resulting in an increased risk of fractures [3]. In Japan, the prevalence of osteoporosis is 3.4% in men and 19.2% in women at the lumber spine (L2–L4) and 12.4% in men and 26.5% in women at the femoral neck [4]. On the other hand, sarcopenia refers to the accelerated decline in skeletal muscle mass plus muscle strength and/or physical performance associated with aging or chronic disease [5, 6]. Recently, the Global Leadership Initiative in Sarcopenia (GLIS) consensus has stated that muscle mass, muscle strength, and muscle-specific strength were accepted as components of sarcopenia, and impaired physical performance was accepted as an outcome rather than a component of sarcopenia [7]. The prevalence of sarcopenia in the Asian region ranges from 5.5% to 25.7% [6]. Sarcopenia is associated with a variety of significant adverse health outcomes, leading to an increased risk of falls and fractures, impaired physical performance, and mortality [5–7].

Recently, bone and muscle have been recognized as interacting tissues due to their close proximity and integrated function for locomotion, and osteoporosis and sarcopenia are thought to share common pathophysiological factors such as physical impairment, release of tissue-specific molecules, and increased inflammatory cytokine activity [8]. It has been reported that sarcopenia is significantly associated with low regional or whole-body BMD [9]. Another previous study provided evidence that the prevalence of sarcopenia was higher in subjects with osteoporosis (29.7%) than those with osteopenia (17.8%) and normal BMD (9.0%) among Japanese women [10]. Therefore, osteoporosis and sarcopenia are likely to occur simultaneously. To convey this close relationship between sarcopenia and osteoporosis, “osteosarcopenia” has recently been defined as a geriatric syndrome characterized by the combined occurrence of osteoporosis or osteopenia and sarcopenia [11]. In older people, osteosarcopenia has been reported to significantly increase the risk of falls, fractures, and death compared with osteoporosis or sarcopenia alone [12, 13]. Therefore, early detection of sarcopenia in patients with osteoporosis is particularly important.

To enable early identification and intervention for people with, or at risk for, sarcopenia, the European Working Group on Sarcopenia in Older People 2 (EWGSOP2) recommends the use of a 5-point self-administered questionnaire known as SARC-F [5]. This questionnaire assesses strength, assistance in walking, rising from a chair, climbing stairs, and falls [14]. Additionally, the 2019 Asian Working Group for Sarcopenia (AWGS 2019) recommends the use of either SARC-F, calf circumference (CC), or their combination (SARC-CalF) for assessment [6].

There have been recent studies comparing the predictive accuracy of these screening tools among community-dwelling older people [15–17]. In terms of overall screening ability, CC has been found to be superior to SARC-F and SARC-CalF for sarcopenia in community-dwelling older people, regardless of age, gender, and cognitive function [15], and regardless of whether the AWGS 2014 or 2019 criteria have been used for sarcopenia diagnosis [16]. However, a scoping review suggests that when SARC-F is combined with CC, its sensitivity is enhanced, leading to an improvement in overall diagnostic performance [17]. Therefore, the results across studies have been inconsistent. On the other hand, Xu *et al*. reported that SARC-CalF appears to be the most appropriate screening tool for sarcopenia in adult patients with type 2 diabetes mellitus [18]. This suggests that different diseases may require different screening tools for predicting sarcopenia. Therefore, the evaluation of screening tools for sarcopenia should be conducted within the context of specific diseases. So far, there are no studies comparing the utility of SARC-F, SARC-CalF, and CC as screening tools for sarcopenia in patients with osteoporosis.

In this study, we aimed to compare the effectiveness of SARC-F, SARC-CalF, and CC as screening tools for sarcopenia in patients with osteoporosis. The findings of this study are anticipated to contribute to the healthy life expectancy of patients with osteoporosis.

## Methods

### Study subjects and design

In this study, consecutive patients who attended the outpatient clinic for Osteoporosis and Sarcopenia at National Hospital Organization (NHO) Kyoto Medical Center (KMC) between July 2019 and June 2021 were retrospectively studied. Patients with artificial hip joints were excluded because the body composition analyzer cannot accurately measure body composition. In addition, patients with missing data on skeletal muscle mass, handgrip strength (HGS), SARC-F, CC, and BMD were excluded. Additionally, patients without a diagnosis of osteoporosis or osteopenia were excluded. According to the previous reports, we estimated the prevalence of the sarcopenia to be 10% [6, 10, 19]. Based on a previous report [20], when the prevalence of sarcopenia is estimated at 10%, a minimum sample size of 200 patients (including 20 patients having sarcopenia) will be required to achieved a minimum power of 80% to detect a change in the percentage value of sensitivity from 50% to 80%, based on a target significance level of 0.05. This minimum sample size is also sufficient to detect a change in the value of specificity from 80% to 90% which will only require a minimum sample of 119 patients (including 12 patients having sarcopenia). Therefore, we collected patients so that at least 200 patients would be included in the analysis. Oral consent to participate was obtained from all patients before study inclusion. Written consent was not acquired because this study is a retrospective study. All patients’ records and information were anonymized and de-identified prior to analysis. This study was approved by the Institutional Ethics Committee of the NHO KMC (approval number 18–076), and this study conformed to the 1964 Helsinki declaration and its later amendments or comparable ethical standards.

### Diagnosis of osteopenia and osteoporosis

BMD was assessed at the lumber spine (L2–L4) and femoral neck using dual-energy X-ray absorptiometry (Discovery WI, Hologic, Waltham, MA, USA). In this study, the presence of osteoporosis and osteopenia was determined based on low BMD at the lumber or femoral neck and/or the occurrence of a fragility fracture, following the guidelines of the Japanese Society for Bone and Mineral Research and the World Health Organization criteria [21, 22]. Briefly, osteoporosis was diagnosed when the BMD was equal to or below 70% or −2.5 standard deviation (SD) of the young adult mean (YAM), or in the presence of a fragility fracture at the lumber spine or proximal femur, or in the presence of another fragility fracture with a BMD below 80% of YAM. Osteopenia was diagnosed when the BMD was equal to or below −1.0 SD but above −2.5 SD of YAM, without meeting the criteria for osteoporosis.

### Body mass index and diagnosis of sarcopenia

All anthropometric measurements were conducted in the morning by a trained technician to ensure accuracy. Height and body weight values were measured to the nearest 0.1 cm and 0.1 kg, respectively. Body mass index (BMI, kg/m^2^) was calculated by dividing the body weight (kg) by the square of the height (m^2^). A multi-frequency segmental body composition analyzer (MC-780A-N, TANITA Co., Ltd., Tokyo, Japan) was used to measure bioelectrical impedance and obtain data on whole body composition. An estimation formula for appendicular skeletal muscle mass in this model has been published, and a previous validation study showed that body composition measured using this device was highly correlated with that measured dual-energy X-ray absorptiometry measurements [23]. The skeletal muscle mass index (SMI, kg/m^2^) was calculated by dividing the appendicular skeletal muscle mass (kg) by the square of the height (m^2^). HGS was measured in the standing position with the arms straight down to the sides. The maximum grip strengths of the right and left hands were measured two times, alternatively with rests as necessary, using a Smedley Spring-Type Dynamometer (Grip-D, Takei Scientific Instruments Co., Ltd., Niigata, Japan) to the nearest 0.1 kg [24–26]. The maximum values of these measurements were used for analyses, as they are less likely to be affected by the number of trials than the means [27, 28]. Sarcopenia was diagnosed based on the EWGSOP2 criteria, that is sarcopenia was confirmed by low SMI and weak HGS [5]. The cutoff values for SMI were set at less than 7.0 kg/m^2^ for men and less than 5.7 kg/m^2^ for women, while the cutoff values for HGS were set at less than 28 kg for men and less than 18 kg for women [6].

### Screening tools for sarcopenia

In the study subjects, the effectiveness of SARC-F, SARC-CalF, and CC as screening tools for sarcopenia was compared. The SARC-F assessment involved a 5-point self-administered questionnaire, with each item having a score range of 0 to 2 and a total score range of 0 to 10 [14]. The Japanese version of SARC-F was used [29], and a score of 4 or higher indicated a positive result for sarcopenia screening [6]. CC was measured to the nearest 0.1 cm in the standing position using a nonelastic tape measure [30]. The tape measure was placed around the calf without compressing the subcutaneous tissue, and the maximal circumference was recorded by moving the tape measure along the length of the calf. The maximum CC values from both legs were used [31], with values below 34 cm for men and 33 cm for women considered positive for sarcopenia screening [6, 30, 31]. SARC-CalF was a composite score combining CC and SARC-F components. It was calculated by adding 10 points to the SARC-F score when the CC measurement fell below the cutoff value [32]. A score of 11 or higher indicated a positive result for sarcopenia screening [6].

### Presence of hypertension, diabetes mellitus, dyslipidemia, and cancer

The presence of hypertension, diabetes mellitus, dyslipidemia, and cancer was determined *via* self-reporting and/or by considering the use of medications for each disease.

### Statistical analysis

Categorical variables were presented as numbers and percentages and analyzed using the χ2 test or Fisher’s exact test, as appropriate. Continuous variables were presented as medians and interquartile range (IQR) and analyzed using the Mann–Whitney *U* test. The diagnostic utility of the three screening tools to predict the presence of sarcopenia was compared using the receiver-operating characteristic (ROC) curves, the area under the ROC curves (AUC), and 95% confidence interval (CI). The AUCs were compared each other using the DeLong method [33]. A larger AUC indicated better overall diagnostic accuracy [34]. A multivariate logistic regression was performed to assess whether the screening tool with the highest AUC and diagnostic potential among the three remained associated with sarcopenia even after adjusting for other sarcopenia-related factors. Comparing between the sarcopenia and non-sarcopenia groups, factors that might influence sarcopenia screening tools other than indices for sarcopenia diagnosis, such as SMI, HGS, and CC, were identified, and these factors were defined as confounders. These analyses were performed using the EZR software (Saitama Medical Center, Jichi Medical University, Saitama, Japan) [35] and the Statistical Package for the Social Sciences software Version 29.0 (SPSS; IBM Corporation, Armonk, NY, USA). All statistical tests were two-tailed, and a *p*-value of less than 0.05 was considered statistically significant.

## Results

### Characteristics of the study population

**Fig 1:**
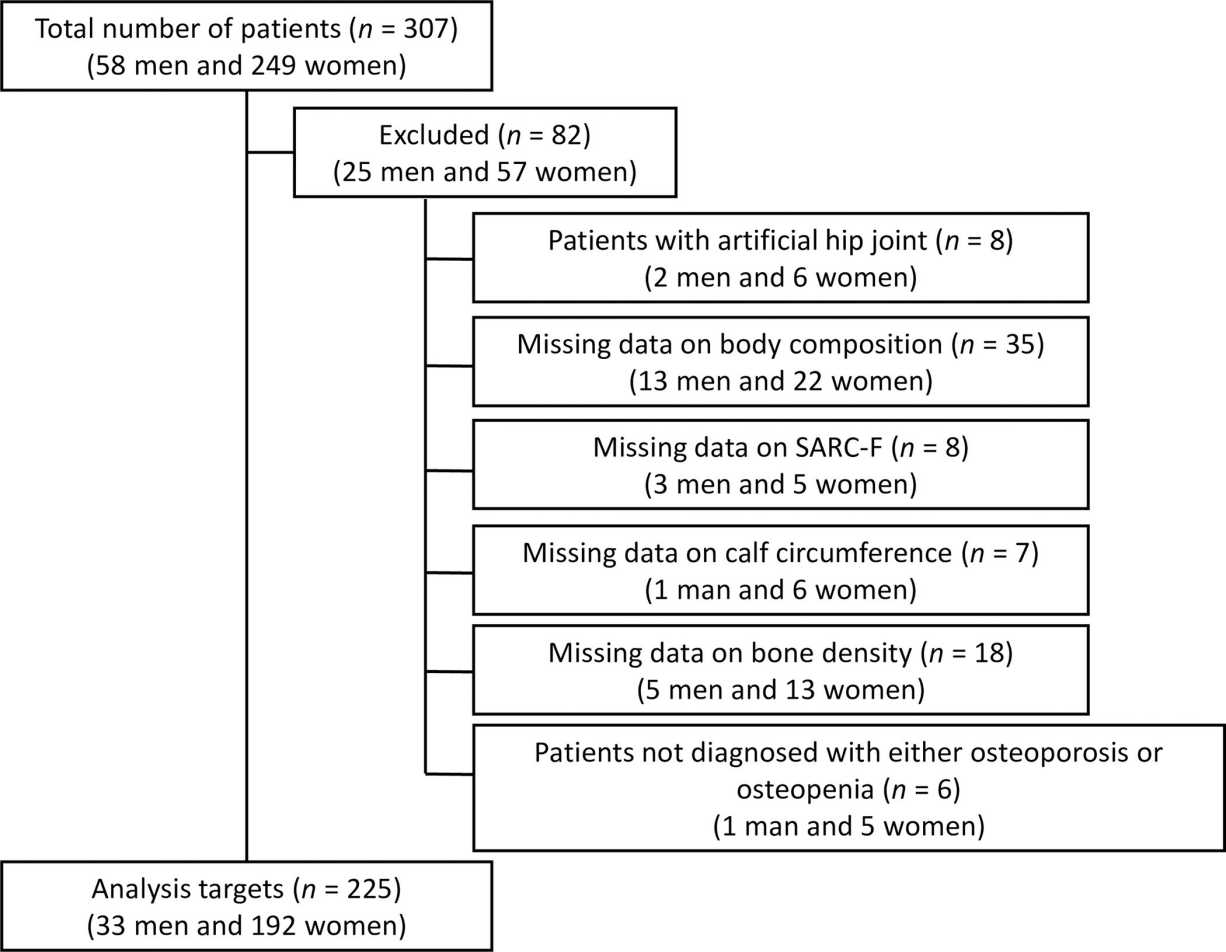
Flowchart for selecting analysis targets.

A total of 307 patients (men/women: 58/249) attended the outpatient clinic for Osteoporosis and Sarcopenia at NHO KMC between July 2019 and June 2021. However, 82 patients who met the exclusion criteria were excluded, and 225 patients (men/women: 33/192) were included in the analysis (Fig 1). The characteristics of the study population are summarized in Table 1. The median age of the patients was 69.0 years (IQR: 61.0–75.0). In this study, the prevalence of sarcopenia was 11.6% (36.4% in men and 7.3% in women). For each screening tool for sarcopenia, the positive rates were 9.8% for SARC-F (12.1% in men and 9.4% in women), 24.4% for SARC-CalF (21.2% in men and 25.0% in women), and 45.3% for CC (45.5% in men and 45.3% in women). A total of 153 patients (68.0%) were receiving osteoporosis treatment (Bisphosphonates/Active vitamin D/Calcium/Selective estrogen receptor modulator/Anti-receptor activator of nuclear factor kappa B ligand antibody/Sclerostin antibody: 44/106/45/26/39/5).

**Table 1:** Characteristics of the study population.

	All population (*n* = 225)	Men (*n* = 33)	Women (*n* = 192)
Age, year (IQR)	69.0 (61.0–75.0)	71.0 (66.0–78.0)	68.0 (61.0–74.3)
BMI, kg/m^2^ (IQR)	21.3 (18.8–23.2)	21.8 (18.8–23.6)	21.2 (18.6–23.2)
SMI, kg/m^2^ (IQR)	6.3 (6.0–6.6)	6.9 (6.4–7.6)	6.2 (5.9–6.5)
HGS, kg (IQR)	23.0 (19.3–26.3)	31.2 (25.9–35.1)	22.1 (19.2–24.9)
CC, cm (IQR)	33.0 (31.2–35.0)	34.0 (32.3–36.4)	33.0 (31.0–34.6)
Sarcopenia, *n* (%)	26 (11.6)	12 (36.4)	14 (7.3)
Lumber bone T score (IQR)	−2.5 (−3.2,−1.5)	−2.3 (−3.1,−0.8)	−2.5 (−3.2,−1.5)
Femoral bone T score (IQR)	−2.4 (−2.8,−2.0)	−2.2 (−2.6,−1.7)	−2.4 (−2.8,−2.0)
Osteoporosis, *n* (%)	203 (90.2)	31 (93.9)	172 (89.6)
Osteopenia, *n* (%)	22 (9.8)	2 (6.1)	20 (10.4)
Vulnerability fracture, *n* (%)	41 (18.2)	10 (30.3)	31 (16.1)
SARC-F test-positive, *n* (%)	22 (9.8)	4 (12.1)	18 (9.4)
SARC-CalF test-positive, *n* (%)	55 (24.4)	7 (21.2)	48 (25.0)
Calf circumference test-positive, *n* (%)	102 (45.3)	15 (45.5)	87 (45.3)
Hypertension, *n* (%)	64 (28.4)	15 (45.5)	49 (25.5)
Diabetes mellitus, *n* (%)	30 (13.3)	9 (27.3)	21 (10.9)
Dyslipidemia, *n* (%)	69 (30.7)	9 (27.3)	60 (31.2)
Cancer, *n* (%)	67 (29.8)	11 (33.3)	56 (29.2)
RA / PMR, *n* (%)	2 (0.9) / 3 (1.3)	0 (0.0) / 1 (3.0)	2 (1.0) / 2 (1.0)
Osteoporosis treatment, *n* (%)	153 (68.0)	22 (66.7)	131 (68.2)

Values are represented by medians with interquartile ranges for continuous variables and by numbers and percentages for categorical variables. *n*, number of patients; IQR, interquartile range; BMI, body mass index; SMI, skeletal muscle mass index; HGS, handgrip strength; CC, calf circumference; RA, rheumatoid arthritis; PMR, polymyalgia rheumatica.

### Comparison of patients with and without sarcopenia

The characteristics of patients with or without sarcopenia are summarized and compared in Table 2. Significant differences were found between the two groups in terms of gender (men/women: 12/14 *vs*. 21/178, *p* < 0.001), age (76.5 years [IQR: 72.3–80.8] *vs*. 68.0 years [IQR: 60.5–74.0], *p* < 0.001), SMI (5.37 kg/m^2^ [IQR: 5.13–5.97] *vs*. 6.33 kg/m^2^ [IQR: 6.04–6.67], *p* < 0.001), HGS (16.9 kg [IQR: 15.2–23.8] *vs*. 23.1 kg [IQR: 20.2–26.4], *p* < 0.001), CC (31.0 cm [IQR: 29.0–32.2] *vs*. 33.3 cm [IQR: 31.5–35.1], *p* < 0.001), femoral bone T score (−2.8 [IQR: −3.3,−2.4] *vs*. −2.3 [IQR: −2.8,−2.0], *p* = 0.008), and the positive rates for each sarcopenia screening tool, including SARC-F test-positive (30.8% *vs*. 7.0%, *p* = 0.001), SARC-CalF test-positive (69.2% *vs*. 18.6%, *p* < 0.001), and CC test-positive (80.8% *vs*. 40.7%, *p* < 0.001). In addition, significant differences were found between the two groups in terms of the complicated rates of hypertension (53.8% *vs*. 25.1%, *p* = 0.005) and diabetes mellitus (38.5% *vs*. 10.1%, *p* < 0.001).

**Table 2:** Comparison of patients with and without sarcopenia.

	Sarcopenia (*n* = 26)	Non-sarcopenia (*n* = 199)	*P*-value
Gender (men/women), *n*	12/14	21/178	<0.001
Age, year (IQR)	76.5 (72.3–80.8)	68.0 (60.5–74.0)	<0.001
BMI, kg/m^2^ (IQR)	19.6 (18.1–22.5)	21.4 (18.8–23.2)	0.173
SMI, kg/m^2^ (IQR)	5.37 (5.13–5.97)	6.33 (6.04–6.67)	<0.001
HGS, kg (IQR)	16.9 (15.2–23.8)	23.1 (20.2–26.4)	<0.001
CC, cm (IQR)	31.0 (29.0–32.2)	33.3 (31.5–35.1)	<0.001
Lumber bone T score (IQR)	−2.3 (−3.1,−2.0)	−2.5 (−3.2,−1.5)	0.790
Femoral bone T score (IQR)	−2.8 (−3.3,−2.4)	−2.3 (−2.8,−2.0)	0.008
Osteoporosis, *n* (%)	26 (100)	177 (88.9)	0.085
Vulnerability fracture, *n* (%)	8 (30.8)	33 (16.6)	0.102
SARC-F test-positive, *n* (%)	8 (30.8)	14 (7.0)	0.001
SARC-CalF test-positive, *n* (%)	18 (69.2)	37 (18.6)	<0.001
Calf circumference test-positive, *n* (%)	21 (80.8)	81 (40.7)	<0.001
Hypertension, *n* (%)	14 (53.8)	50 (25.1)	0.005
Diabetes mellitus, *n* (%)	10 (38.5)	20 (10.1)	<0.001
Dyslipidemia, *n* (%)	11 (42.3)	58 (29.1)	0.181
Cancer, *n* (%)	8 (30.8)	59 (29.6)	1.000

Values are represented by medians and interquartile ranges for continuous variables and by numbers and percentages for categorical variables. *n*, number of patients; IQR, interquartile range; BMI, body mass index; SMI, skeletal muscle mass index; HGS, handgrip strength; CC, calf circumference

### Comparison of the studied screening tools for sarcopenia

Table 3 presents the performance characteristics of SARC-F, SARC-CalF, and CC in sarcopenia screening. CC had the highest sensitivity compared to SARC-F and SARC-CalF (80.8% *vs*. 30.8% and 69.2%). On the other hand, SARC-F had the highest specificity compared to SARC-CalF and CC (93.0% *vs*. 81.4% and 59.3%). The AUC of SARC-CalF was significantly higher than those of SARC-F and CC in women. When men and women were analyzed together, the AUC of SARC-CalF (0.753, 95% CI: 0.659–0.848) was higher than those of SARC-F (0.619, 95% CI: 0.526–0.711) and CC (0.700, 95% CI: 0.616–0.785), suggesting that SARC-CalF has better diagnostic accuracy for sarcopenia than SARC-F and CC.

**Table 3:** Performance characteristics of SARC-F, SARC-CalF, and CC for sarcopenia screening in men and women.

		Sensitivity, %	Specificity, %	PPV, %	NPV, %	AUC
Total (*n* = 225)	SARC-F	30.8 (14.3–51.8)	93.0 (88.5–96.1)	36.4 (17.2–59.3)	91.1 (86.3–94.7)	0.619 (0.526–0.711)
	SARC-CalF	69.2 (48.2–85.7)	81.4 (75.3–86.6)	32.7 (20.7–46.7)	95.3 (90.9–97.9)	0.753 (0.659–0.848)
	CC	80.8 (60.6–93.4)	59.3 (52.1–66.2)	20.6 (13.2–29.7)	95.9 (90.8–98.7)	0.700 (0.616–0.785)
Men (*n* = 33)	SARC-F	25.0 (5.5–57.2)	95.2 (76.2–99.9)	75.0 (19.4–99.4)	69.0 (49.2–84.7)	0.601 (0.465–0.737)
	SARC-CalF	41.7 (15.2–72.3)	90.5 (69.6–98.8)	71.4 (29.0–96.3)	73.1 (52.2–88.4)	0.661 (0.501–0.820)
	CC	66.7 (34.9–90.1)	66.7 (43.0–85.4)	53.3 (26.6–78.7)	77.8 (52.4–93.6)	0.667 (0.493–0.840)
Women (*n* = 192)	SARC-F	35.7 (12.8–64.9)	92.7 (87.8–96.1)	27.8 (9.7–53.5)	94.8 (90.4–97.6)	0.642 (0.510–0.774) ^a^
	SARC-CalF	92.9 (66.1–99.8)	80.3 (73.7–85.9)	27.1 (15.3–41.8)	99.3 (96.2–100)	0.866 (0.790–0.942)
	CC	92.9 (66.1–99.8)	58.4 (50.8–65.8)	14.9 (8.2–24.2)	99.0 (94.8–100)	0.756 (0.678–0.835) ^a^

Values within parentheses are the 95% confidential intervals. *n*, number of patients; CC, calf circumference; PPV, positive predictive value; NPV, negative predictive value; AUC, area under the curve

^a^ Significantly different with SARC-CalF (*p* < 0.05)

### A multivariate logistic regression for the prediction of sarcopenia

Comparison of the sarcopenia and non-sarcopenia groups showed that, other than the indices for sarcopenia diagnosis, gender, age, femoral T-score, presence of hypertension, and presence of diabetes mellitus were significantly different (Table 2). Then, using these as explanatory variables, a multivariate analysis was performed to determine whether SARC-CalF, which had the highest AUC, was independently associated with sarcopenia (Table 4). After excluding these confounders, SARC-CalF was found to be independently related to sarcopenia (odds ratio: 14.80, 95% CI: 3.83–57.30, *p* < 0.001). In addition, we also examined the relationship between SARC-F or CC and sarcopenia. Then, we found that CC was related to sarcopenia even after adjusting for other covariates (odds ratio: 7.37, 95% CI: 2.24–24.30, *p* < 0.001) but not SARC-F (S1 and S2 Tables).

**Table 4:** A multivariate logistic regression for the prediction of sarcopenia with SARC-CalF.

	Odds ratio	95% CI	*P*-value
SARC-CalF test-positive	14.80	3.83–57.30	<0.001
Age, year	1.07	1.00–1.14	0.055
Gender (Men)	22.10	5.14–95.00	<0.001
Femoral bone T score	0.50	0.23–1.10	0.089
Diabetes mellitus	3.87	1.13–13.30	0.031
Hypertension	1.42	0.42–4.77	0.574

Sarcopenia as objective variable in the multivariate logistic regression analysis. Explanatory variables included in the multivariate logistic regression analysis are SARC-CalF test-positive (i.e. SARC-CalF score is 11 or higher), age, gender, femoral bone T score, presence of diabetes mellitus, and presence of hypertension. 95% CI, 95% confidence intervals.

## Discussion

To the best of our knowledge, this study is the first to demonstrate that SARC-CalF is a more effective screening tool for sarcopenia in patients with osteoporosis than SARC-F and CC. Furthermore, SARC-CalF was found to be independently related to sarcopenia, even after accounting for other confounders. The presence of osteosarcopenia has significant implications for overall health outcomes. Therefore, it is crucial to identify screening tools that can easily and accurately detect sarcopenia in patients with osteoporosis. The findings of this study have the potential to improve the healthy life expectancy of patients with osteoporosis.

In this study, the prevalence of sarcopenia in patients with osteoporosis was found to be 11.6%. A review of epidemiological studies conducted in older adults from Asian countries reported a prevalence of sarcopenia ranging from 5.5% to 25.7% [6]. In a study specifically focused on patients with osteoporosis, the prevalence of sarcopenia was reported as 10.8% in patients with low BMD [19]. Another study conducted on Japanese women observed a higher prevalence of sarcopenia in subjects with osteoporosis (29.7%) than in those with osteopenia (17.8%) and normal BMD (9.0%) [10]. Although this study did not examine the prevalence of sarcopenia in individuals without osteoporosis, the prevalence of sarcopenia in patients with osteoporosis appeared to be consistent with previous studies.

In this study conducted on patients with osteopenia and osteoporosis, CC had the highest sensitivity compared to SARC-F and SARC-CalF. In addition, SARC-F had the highest specificity compared to SARC-CalF and CC. A previous study comparing the performance of screening tools for sarcopenia among community-dwelling older adults reported a sensitivity of 17.9% and a specificity of 93.7% for SARC-F, a sensitivity of 47.5% and a specificity of 92.0% for SARC-CalF, and a sensitivity of 81.4% and a specificity of 77.0% for CC [15]. Although the target population in our study was different, our findings were consistent with this previous study.

SARC-F and CC have different characteristics as screening tools for sarcopenia. SARC-F is a self-administered questionnaire that primarily reflects physical ability [14, 29]. However, due to its subjective nature, SARC-F may have limited screening ability to exclude sarcopenia [36]. Especially, men tend to select answers that evaluate their physical ability more favorably and lightly than women [37]. On the other hand, CC is positively correlated with appendicular skeletal muscle mass and SMI [30]. Although CC is an objective indicator, it can be influenced by factors such as obesity and edema, which may mask the presence of sarcopenia [17, 38]. Therefore, caution should be taken when measuring CC in the presence of these conditions. In this study, CC was considered to accurately reflect skeletal muscle mass as there were few obese or edematous patients with osteoporosis. Considering these factors, the combination of SARC-F, which reflects physical ability, and CC, which reflects skeletal muscle mass, in SARC-CalF was deemed to be more useful than using SARC-F and CC alone in patients with osteoporosis.

In general, a higher AUC indicates better overall performance of a diagnostic test [34]. Therefore, the performance of a screening tool is evaluated using the AUC. In this study conducted on patients with osteoporosis, SARC-CalF had the highest AUC compared to SARC-F and CC (0.753 *vs*. 0.619 and 0.700; Table 3). On the other hand, in the previous study conducted on community-dwelling older adults, CC had the highest AUC compared to SARC-F and SARC-CalF (0.80 *vs*. 0.56 and 0.70) [15]. This study excluded people taking medications that affect body composition, such as diuretics and glucocorticoids, and people with clinically visible edema, suggesting that it targeted people with relatively preserved physical ability compared with our osteoporotic patients [13]. Such differences in the target population among studies may cause the discrepancy in the most effective screening tool for sarcopenia. As patients with both osteoporosis and sarcopenia are at a high risk of falls and fractures [12], it may be more appropriate to use SARC-F, which reflects physical ability. Taken together, we assume that SARC-CalF, in combination with SARC-F and CC, provides a better balance and higher AUC than SARC-F or CC alone as a screening tool for sarcopenia in patients with osteoporosis.

In this study, we compared the characteristics of patients with or without sarcopenia and observed significant differences in the presence of diabetes mellitus and hypertension, as well as in each sarcopenia screening tool, gender, age, and femoral bone T score. A previous systematic review and meta-analysis has also demonstrated an association between type 2 diabetes mellitus and an increased risk of sarcopenia, with some pathophysiological mechanisms underlying this association [39]. Furthermore, sarcopenia has been found to be associated with hypertension in another systematic review and meta-analysis [40]. Since the shared mechanism of chronic inflammation and catabolic cytokine production is the most accepted mechanism of sarcopenia and a major risk factor for chronic diseases such as hypertension, sarcopenia and hypertension may be associated through these shared mechanisms [40]. Therefore, the factors associated with sarcopenia in this study are consistent with those reported in previous studies.

This study has several limitations that should be acknowledged. First, it had a retrospective single-center-based design, which may introduce bias in the ratio of men to women and the severity of osteoporosis, and the exact data on the duration of sarcopenia exposure could not be addressed. Second, the history of fragility fractures relied on self-reporting, and other fragility fractures beyond lumber and femoral neck fractures were not assessed, potentially resulting in missing some patients with osteoporosis. Third, in this study, sarcopenia was diagnosed based on the EWGSOP2 criteria [5], so patients with low physical performance, low muscle mass, and high handgrip strength were included in the non-sarcopenia group. If sarcopenia is diagnosed based on the AWGS2019 criteria [6], another analysis will be needed. Fourth, in this study, we excluded patients with artificial hip joints because we could not evaluate body composition accurately. However, patients with artificial hip joints due to fragility fractures are diagnosed as osteoporosis. As for osteoporotic patients with artificial hip joints, another analysis will be need in the future. Fifth, this study did not examine chronic kidney disease, endocrine disease, exercise and nutritional status, or frailty, which may affect sarcopenia and osteoporosis [8, 41–43]. Finally, the effects of medications taken by the study patients were not considered, although certain medications for osteoporosis, hypertension, and diabetes mellitus have been suggested to affect skeletal muscles [44]. Validation studies with larger sample sizes are needed to address these limitations.

### Conclusion

In conclusion, while SARC-F, SARC-CalF, and CC are all simple and useful screening tools for the early detection of sarcopenia, SARC-CalF appears to be more effective than SARC-F and CC as a screening tool for sarcopenia in patients with osteoporosis. Further research is needed to validate the utility of SARC-CalF in various populations.

## Supporting information

S1 Table A multivariate logistic regression for the prediction of sarcopenia with SARC-F. (DOCX)

S2 Table A multivariate logistic regression for the prediction of sarcopenia with CC. (DOCX)

S1 Dataset (XLSX)

## References

[1] Hébert R. Functional decline in old age. *CMAJ* 1997;157(8):1037–45. 9347774 PMC1228259

[2] Sepúlveda-Loyola W, Phu S, Bani Hassan E, Brennan-Olsen SL, Zanker J, Vogrin S, et al. The joint occurrence of osteoporosis and sarcopenia (osteosarcopenia): definitions and characteristics. *J Am Med Dir Assoc* 2020;21(2):220–225. doi: 10.1016/j.jamda.2019.09.005 31669290

[3] Kanis JA. Diagnosis of osteoporosis and assessment of fracture risk. *Lancet* 2002;359(9321):1929–1936. doi: 10.1016/S0140-6736(02)08761-5 12057569

[4] Yoshimura N, Muraki S, Oka H, Mabuchi A, En-Yo Y, Yoshida M, et al. Prevalence of knee osteoarthritis, lumbar spondylosis, and osteoporosis in Japanese men and women: the research on osteoarthritis/osteoporosis against disability study. *J Bone Miner Metab* 2009;27(5):620–628. doi: 10.1007/s00774-009-0080-8 19568689

[5] Cruz-Jentoft AJ, Bahat G, Bauer J, Boirie Y, Bruyère O, Cederholm T, et al; Writing Group for the European Working Group on Sarcopenia. Older People 2 (EWGSOP2), and the extended Group for EWGSOP2. Sarcopenia: revised European consensus on definition and diagnosis. *Age Ageing* 2019;48(1):16–31. doi: 10.1093/ageing/afy169 30312372 PMC6322506

[6] Chen LK, Woo J, Assantachai P, Auyeung TW, Chou MY, Iijima K, et al. Asian Working Group for sarcopenia: 2019 consensus update on sarcopenia diagnosis and treatment. *J Am Med Dir Assoc* 2020;21(3):300–307.e2. doi: 10.1016/j.jamda.2019.12.012 32033882

[7] Kirk B, Cawthon PM, Arai H, Ávila-Funes JA, Barazzoni R, Bhasin S, et al. The Conceptual Definition of Sarcopenia: Delphi Consensus from the Global Leadership Initiative in Sarcopenia (GLIS). *Age Ageing* 2024;53(3):afae052. doi: 10.1093/ageing/afae052 38520141 PMC10960072

[8] Greco EA, Pietschmann P, Migliaccio S. Osteoporosis and sarcopenia increase frailty syndrome in the elderly. *Front Endocrinol* 2019;10:255. doi: 10.3389/fendo.2019.00255 31068903 PMC6491670

[9] Ning HT, Du Y, Zhao LJ, Tian Q, Feng H, Deng HW. Racial and gender differences in the relationship between sarcopenia and bone mineral density among older adults. *Osteoporos Int* 2021;32(5):841–851. doi: 10.1007/s00198-020-05744-y 33231702 PMC8044008

[10] Miyakoshi N, Hongo M, Mizutani Y, Shimada Y. Prevalence of sarcopenia in Japanese women with osteopenia and osteoporosis. *J Bone Miner Metab* 2013;31(5):556–561. doi: 10.1007/s00774-013-0443-z 23515924

[11] Huo YR, Suriyaarachchi P, Gomez F, Curcio CL, Boersma D, Muir SW, et al. Phenotype of osteosarcopenia in older individuals with a history of falling. *J Am Med Dir Assoc* 2015;16(4):290–295. doi: 10.1016/j.jamda.2014.10.018 25512216

[12] Teng Z, Zhu Y, Teng Y, Long Q, Hao Q, Yu X, et al. The analysis of osteosarcopenia as a risk factor for fractures, mortality, and falls. *Osteoporos Int* 2021;32(11):2173–2183. doi: 10.1007/s00198-021-05963-x 33877382

[13] Drey M, Sieber CC, Bertsch T, Bauer JM, Schmidmaier R, FiAT intervention group. Osteosarcopenia is more than sarcopenia and osteopenia alone. *Aging Clin Exp Res* 2016;28(5):895–899. doi: 10.1007/s40520-015-0494-1 26563287

[14] Malmstrom TK, Morley JE. SARC-F: a simple questionnaire to rapidly diagnose sarcopenia. *J Am Med Dir Assoc* 2013;14(8):531–532. doi: 10.1016/j.jamda.2013.05.018 23810110

[15] Mo YH, Zhong J, Dong X, Su YD, Deng WY, Yao XM, et al. Comparison of three screening methods for sarcopenia in community-dwelling older persons. *J Am Med Dir Assoc* 2021;22(4):746–750.e1. doi: 10.1016/j.jamda.2020.05.041 32669238

[16] Ito A, Ishizaka M, Kobayashi K, Sawaya Y, Hara T, Nagasaka Y, et al. Changes in the screening efficacy of lower calf circumference, SARC-F score, and SARC-calf score following update from AWGS 2014 to 2019 sarcopenia diagnostic criteria in community-dwelling older adults. *J Phys Ther Sci* 2021;33(3):241–245. doi: 10.1589/jpts.33.241 33814711 PMC8012190

[17] Mohd Nawi SN, Khow KS, Lim WS, Yu SC. Screening tools for sarcopenia in community-dwellers: a scoping review. *Ann Acad Med Singap* 2019;48(7):201–216. 31495866

[18] Xu Z, Zhang P, Chen Y, Jiang J, Zhou Z, Zhu H. Comparing SARC-CalF with SARC-F for screening sarcopenia in adults with type 2 diabetes mellitus. *Front Nutr* 2022;9:803924. doi: 10.3389/fnut.2022.803924 35433779 PMC9009513

[19] Pourhassan M, Buehring B, Stervbo U, Rahmann S, Mölder F, Rütten S, et al. Osteosarcopenia, an asymmetrical overlap of two connected syndromes: data from the OsteoSys study. *Nutrients* 2021;13(11):3786. doi: 10.3390/nu13113786 34836043 PMC8618221

[20] Bujang MA, Adnan TH. Requirements for Minimum Sample Size for Sensitivity and Specificity Analysis. J Clin Diagn Res 2016;10(10):YE01–YE06. doi: 10.7860/JCDR/2016/18129.8744 27891446 PMC5121784

[21] Soen S, Fukunaga M, Sugimoto T, Sone T, Fujiwara S, Endo N, et al. Diagnostic criteria for primary osteoporosis: year 2012 revision Japanese society for bone and mineral research and Japan osteoporosis society joint review committee for the revision of the diagnostic criteria for primary osteoporosis. *J Bone Miner Metab* 2013;31(3):247–257. doi: 10.1007/s00774-013-0447-8 23553500

[22] Kanis JA, Melton LJ, Christiansen C, Johnston CC, Khaltaev N. The diagnosis of osteoporosis. *J Bone Miner Res* 1994;9(8):1137–1141. doi: 10.1002/jbmr.5650090802 7976495

[23] Yamada Y, Nishizawa M, Uchiyama T, Kasahara Y, Shindo M, Miyachi M, et al. Developing and Validating an Age-Independent Equation Using Multi-Frequency Bioelectrical Impedance Analysis for Estimation of Appendicular Skeletal Muscle Mass and Establishing a Cutoff for Sarcopenia. *International journal of environmental research and public health* 2017;14(7):809. doi: 10.3390/ijerph14070809 28753945 PMC5551247

[24] Cooper R, Hardy R, Bann D, Aihie Sayer A, Ward KA, Adams JE, Kuh D. Body mass index from age 15 years onwards and muscle mass, strength, and quality in early old age: findings from the MRC National Survey of Health and Development. *J Gerontol A Biol Sci Med Sci* 2014;69(10):1253–1259. doi: 10.1093/gerona/glu039 24682351 PMC4158414

[25] Lees MJ, Wilson OJ, Hind K, Ispoglou T. Muscle quality as a complementary prognostic tool in conjunction with sarcopenia assessment in younger and older individuals. *Eur J Appl Physiol* 2019;119(5):1171–1181. doi: 10.1007/s00421-019-04107-8 30806780 PMC6469623

[26] Akamatsu Y, Kusakabe T, Arai H, Yamamoto Y, Nakao K, Ikeue K, et al. Phase angle from bioelectrical impedance analysis is a useful indicator of muscle quality. *J Cachexia Sarcopenia Muscle* 2022;13(1):180–189. doi: 10.1002/jcsm.12860 34845859 PMC8818694

[27] Dodds RM, Syddall HE, Cooper R, Benzeval M, Deary IJ, Dennison EM, et al. Grip strength across the life course: normative data from twelve British studies. *PLoS One* 2014;9(12):e113637. doi: 10.1371/journal.pone.0113637 25474696 PMC4256164

[28] Roberts HC, Denison HJ, Martin HJ, Patel HP, Syddall H, Cooper C, et al. A review of the measurement of grip strength in clinical and epidemiological studies: towards a standardised approach. *Age Ageing* 2011;40(4):423–429. doi: 10.1093/ageing/afr051 21624928

[29] Ida S, Murata K, Nakadachi D, Ishihara Y, Imataka K, Uchida A, et al. Development of a Japanese version of the SARC-F for diabetic patients: an examination of reliability and validity. *Aging Clin Exp Res* 2017;29(5):935–942. doi: 10.1007/s40520-016-0668-5 27832470 PMC6702187

[30] Kawakami R, Murakami H, Sanada K, Tanaka N, Sawada SS, Tabata I, et al. Calf circumference as a surrogate marker of muscle mass for diagnosing sarcopenia in Japanese men and women. *Geriatr Gerontol Int* 2015;15(8):969–976. doi: 10.1111/ggi.12377 25243821

[31] Hwang AC, Liu LK, Lee WJ, Peng LN, Chen LK. Calf circumference as a screening instrument for appendicular muscle mass measurement. *J Am Med Dir Assoc* 2018;19(2):182–184. doi: 10.1016/j.jamda.2017.11.016 29306606

[32] Mo Y, Dong X, Wang XH. Screening accuracy of SARC-F combined with calf circumference for sarcopenia in older adults: a diagnostic meta-analysis. *J Am Med Dir Assoc* 2020;21(2):288–289. doi: 10.1016/j.jamda.2019.09.002 31672568

[33] DeLong ER, DeLong DM, Clarke-Pearson DL. Comparing the areas under two or more correlated receiver operating characteristic curves: a nonparametric approach. *Biometrics* 1988;44(3):837–845. 3203132

[34] Park SH, Goo JM, Jo CH. Receiver operating characteristic (ROC) curve: practical review for radiologists. *Korean J Radiol* 2004;5(1):11–18. doi: 10.3348/kjr.2004.5.1.11 15064554 PMC2698108

[35] Kanda Y. Investigation of the freely available easy-to-use software ‘EZR’ for medical statistics. *Bone Marrow Transplant* 2013;48(3):452–458. doi: 10.1038/bmt.2012.244 23208313 PMC3590441

[36] Ida S, Kaneko R, Murata K. SARC-F for screening of sarcopenia among older adults: a meta-analysis of screening test accuracy. *J Am Med Dir Assoc* 2018;19(8):685–689. doi: 10.1016/j.jamda.2018.04.001 29778639

[37] Kim M, Yabushita N, Tanaka K. Exploring effective items of physical function in slow walking speed and self-reported mobility limitation in community-dwelling older adults. *Geriatr Gerontol Int* 2012;12(1):50–58. doi: 10.1111/j.1447-0594.2011.00726.x 21729226

[38] Yang M, Lu J, Jiang J, Zeng Y, Tang H. Comparison of four sarcopenia screening tools in nursing home residents. *Aging Clin Exp Res* 2019;31(10):1481–1489. doi: 10.1007/s40520-018-1083-x 30539542

[39] Anagnostis P, Gkekas NK, Achilla C, Pananastasiou G, Taouxidou P, Mitsiou M, et al. Type 2 diabetes mellitus is associated with increased risk of sarcopenia: a systematic review and meta-analysis. *Calcif Tissue Int* 2020;107(5):453–463. doi: 10.1007/s00223-020-00742-y 32772138

[40] Bai T, Fang F, Li F, Ren Y, Hu J, Cao J. Sarcopenia is associated with hypertension in older adults: a systematic review and meta-analysis. *BMC Geriatr* 2020; 20(1):279. doi: 10.1186/s12877-020-01672-y 32762638 PMC7409686

[41] Rashid A, Chaudhary HS, Suetta C, Hansen D. Sarcopenia and risk of osteoporosis, falls and bone fractures in patients with chronic kidney disease: A systematic review. *PLoS One* 2022;17(1):e0262572. doi: 10.1371/journal.pone.0262572 35061818 PMC8782402

[42] Kirk B, Zanker J, Duque G. Osteosarcopenia: epidemiology, diagnosis, and treatment-facts and numbers. *J Cachexia Sarcopenia Muscle* 2020;11(3):609–618. doi: 10.1002/jcsm.12567 32202056 PMC7296259

[43] Papadopoulou SK, Papadimitriou K, Voulgaridou G, Georgaki E, Tsotidou E, Zantidou O, et al. Exercise and nutrition impact on osteoporosis and sarcopenia–The incidence of osteosarcopenia: A narrative review. *Nutrients* 2021;13(12):4499. doi: 10.3390/nu13124499 34960050 PMC8705961

[44] Campins L, Camps M, Riera A, Pleguezuelos E, Yebenes JC, Serra-Prat M. Oral drugs related with muscle wasting and sarcopenia. *A review Pharmacology* 2017;99(1–2):1–8. doi: 10.1159/000448247 27578190

