# Supplementary material for: Comparison of the utility of SARC-F, SARC-CalF, and calf circumference as screening tools for sarcopenia in patients with osteoporosis
Source: PLoS One. 2024 Oct 22;19(10):e0310401. doi: 10.1371/journal.pone.0310401 (PMC11495547; doi:10.1371/journal.pone.0310401)
Supplement: S1 Table — (DOCX) [file pone.0310401.s001.docx]

**S1 Table.** **A multivariate logistic regression for the prediction of sarcopenia with SARC-F**

|  | Odds ratio | 95% CI | *P*-value |
| --- | --- | --- | --- |
| SARC-F test-positive | 3.21 | 0.90–11.40 | 0.072 |
| Age, year | 1.07 | 1.00–1.14 | 0.040 |
| Gender (Men) | 10.90 | 3.51–33.90 | <0.001 |
| Femoral bone T score | 0.37 | 0.17–0.79 | 0.010 |
| Diabetes mellitus | 3.48 | 1.14–10.60 | 0.028 |
| Hypertension | 1.69 | 0.57–5.01 | 0.345 |

Sarcopenia as objective variable in the multivariate logistic regression analysis. Explanatory variables included in the multivariate logistic regression analysis are SARC-F test-positive (i.e. SARC-F score is 4 or higher), age, gender, femoral bone T score, presence of diabetes mellitus, and presence of hypertension. 95% CI, 95% confidence intervals
