# Supplementary material for: Comparison of the utility of SARC-F, SARC-CalF, and calf circumference as screening tools for sarcopenia in patients with osteoporosis
Source: PLoS One. 2024 Oct 22;19(10):e0310401. doi: 10.1371/journal.pone.0310401 (PMC11495547; doi:10.1371/journal.pone.0310401)
Supplement: S2 Table — (DOCX) [file pone.0310401.s002.docx]

**S2 Table. A multivariate logistic regression for the prediction of sarcopenia with CC**

|  | Odds ratio | 95% CI | *P*-value |
| --- | --- | --- | --- |
| CC test-positive | 7.37 | 2.24–24.30 | 0.001 |
| Age, year | 1.08 | 1.00–1.16 | 0.038 |
| Gender (Men) | 13.10 | 3.76–45.90 | <0.001 |
| Femoral bone T score | 0.38 | 0.18–0.82 | 0.014 |
| Diabetes mellitus | 4.19 | 1.26–13.90 | 0.020 |
| Hypertension | 1.85 | 0.58–5.88 | 0.299 |

Sarcopenia as objective variable in the multivariate logistic regression analysis. Explanatory variables included in the multivariate logistic regression analysis are CC test-positive (i.e. CC values below 34 cm for men and 33 cm for women), age, gender, femoral bone T score, presence of diabetes mellitus, and presence of hypertension. 95% CI, 95% confidence intervals; CC, calf circumference
